# Robust Detection and Localization of Image Copy-Move Forgery Using Multi-Feature Fusion

**DOI:** 10.3390/jimaging12020075

**Published:** 2026-02-10

**Authors:** Kaiqi Lu, Qiuyu Zhang

**Affiliations:** School of Computer Science and Artificial Intelligence, Lanzhou University of Technology, Lanzhou 730050, China; lukq_beyond@163.com

**Keywords:** image forensics, copy-move forgery detection, multi-feature fusion, lightweight multi-layer perceptron decoder, dual-branch feature extractor

## Abstract

Copy-move forgery detection (CMFD) is a crucial image forensics analysis technique. The rapid development of deep learning algorithms has led to impressive advancements in CMFD. However, existing models suffer from two key limitations: Their feature fusion modules insufficiently exploit the complementary nature of features from the RGB domain and noise domain, resulting in suboptimal feature representations. During decoding, they simply classify pixels as authentic or forged, without aggregating cross-layer information or integrating local and global attention mechanisms, leading to unsatisfactory detection precision. To overcome these limitations, a robust detection and localization approach to image copy-move forgery using multi-feature fusion is proposed. Firstly, a Multi-Feature Fusion Network (MFFNet) was designed. Within its feature fusion module, features from both the RGB domain and noise domain were fused to enable mutual complementarity between distinct characteristics, yielding richer feature information. Then, a Lightweight Multi-layer Perceptron Decoder (LMPD) was developed for image reconstruction and forgery localization map generation. Finally, by aggregating information from different layers and combining local and global attention mechanisms, more accurate prediction masks were obtained. The experimental results demonstrate that the proposed MFFNet model exhibits enhanced robustness and superior detection and localization performance compared to existing methods when faced with JPEG compression, noise addition, and resizing operations.

## 1. Introduction

With the widespread adoption of editing tools like Adobe Photoshop, copy-move forgery has become one of the most common image manipulation techniques. It involves copying a local region of an image and pasting it elsewhere within the same image to conceal or add content. Because the inherent attributes—such as lighting and noise—of the tampered region remain consistent with the source image, this type of forgery is often difficult to detect visually. It poses a significant threat to the authenticity of images used in critical fields like documentary journalism and forensic evidence [1,2,3]. However, in splicing forgery, the added content is obtained from an unrelated image, while, in CMF, it is sourced from the target image itself. Inpainting forgery involves removing regions from a real image (e.g., concealing objects) and filling the space with newly estimated pixels from the background. Sometimes, post-processing operations, such as rotation, translation, scaling, and smoothing, are applied alongside these forgery techniques to enhance the realism of the forged images.

In recent years, image forgery detection, a technique that classifies and locates tampered areas in digital images, has become a popular research field, with widespread applications in news, diverse scientific fields, security and surveillance, and industrial applications. Based on the characteristics of forgery, various cues, such as blur type inconsistency [4], JPEG compression artifacts [5], noise inconsistency [6], shadow and illumination inconsistency [7], and edge inconsistency [8], have been extensively studied as the foundation for image forgery detection over the past few decades. These studies rely on specific assumptions that the forged and non-forged regions differ. With the superior performance of deep learning in various computer vision tasks, deep learning has also shown promising results in image forgery detection, such as convolutional neural networks (CNNs) detecting image forgery [9,10]. Forgery in real-life scenarios is more complex, as malicious forgers often employ various manipulation techniques to conceal the forgery, which poses significant challenges to the CMFD task. Therefore, designing an effective image forgery detection scheme to detect and localize forged areas is crucial and challenging.

**Figure 1:**
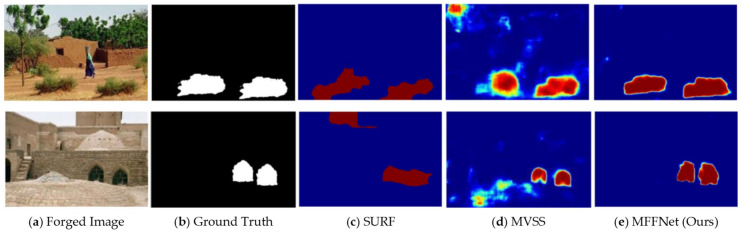
Visual comparison between existing CMFD methods and the proposed approach under challenging scenarios. (**a**) Forged Image, (**b**) Ground Truth, (**c**) SURF, (**d**) MVSS, (**e**) MFFNet (Ours).

Current research on CMFD methods primarily focuses on two broad categories: traditional algorithms and deep learning-based approaches. Traditional algorithms include keypoint-based methods [11] and block-based methods [12]. Block-based methods identify forged areas by matching features extracted from overlapping image blocks. However, these methods are computationally intensive. To reduce computational complexity, researchers have proposed keypoint-based methods. Additionally, there are methods that integrate block and keypoint features. A key limitation of these traditional algorithms is their reliance on predefined features, which hinders their ability to generalize effectively. With the application of deep learning-based approaches in computer vision tasks, many deep learning-based CMFD [13] solutions have been proposed. For example, CMFD methods that involve feature extraction, feature fusion, and decoding modules working together to produce binary masks [13,14,15] have improved CMFD detection performance and localization accuracy. However, these methods fail to fully utilize the extracted feature information, resulting in lower detection performance or suboptimal accuracy. The feature fusion modules in existing models do not integrate feature information from two distinct domains—RGB and noise views—preventing complementary features from being utilized and resulting in lower-quality feature information [14,15]. Furthermore, the decoding stage simply classifies the pixels in the image into real and forged categories, without aggregating information from different layers or incorporating both local and global attention mechanisms [16,17], thus limiting higher precision in forgery detection. As illustrated in Figure 1, a visual comparison is presented among the traditional SURF-based method, the deep learning-based MVSS approach, and the proposed method. Existing CMFD methods often suffer from false positives or inaccurate localization in complex scenarios, such as large homogeneous regions or images subjected to post-processing attacks.

To overcome the aforementioned limitations, we propose a robust detection and localization approach to image copy-move forgery using multi-feature fusion. This method designs a novel detection and localization network (MFFNet), which includes a feature extraction module (DBET encoder), a Concat module, a two-stage feature fusion module (TSFFM), and a Lightweight Multi-layer Perceptron Decoder (LMPD), achieving copy-move forgery detection and localization via the generated prediction masks. The main contributions of this study are as follows:

(1) A Multi-Feature Fusion Network (MFFNet) was designed, where the feature fusion module integrates feature information from both the RGB and noise domains, enabling complementarity between different features to obtain richer feature information, thereby enhancing the recognition ability for complex forgeries and the effectiveness of feature utilization.

(2) A LMPD decoder was designed for image reconstruction and the generation of forgery detection maps. By aggregating information from different layers and combining local and global attention mechanisms, it analyzes forgery traces more comprehensively, improving the robustness and generalization capability of the model.

(3) A deep CMFD framework incorporating a dual-branch feature extractor, a two-stage feature fusion module, and a lightweight multi-layer perceptron decoder is introduced, leveraging the advantages of modern deep CMFD models. Extensive experiments demonstrate that the proposed method significantly outperforms existing comparative algorithms in terms of robustness and exhibits strong generalization to various complex copy-move forgeries.

The remainder of this paper is organized as follows. Section 2 reviews related work. Section 3 provides a detailed explanation of the underlying principles of the copy-move forgery detection and localization method. Section 4 presents the experimental results and analysis. Finally, Section 5 concludes this paper.

## 2. Related Work

Edit-based features left in the image help identify regions with CMF. Current CMFD techniques can be categorized into three types: block-based CMFD methods, keypoint-based CMFD methods, and deep learning-based CMFD methods.

### 2.1. Block-Based CMFD

Generally, in block-based methods, the input image is first divided into overlapping and non-overlapping blocks. Then, feature vectors are extracted from these blocks. Additionally, during the feature-matching phase, features are sorted according to an appropriate data structure to find similar pairs and, finally, the forged regions are located. The first technique for block-based repeated region detection was proposed in [18], which used the DCT algorithm to extract features from each block and compared these features. Additionally, the work by Mahmood et al. [19] determined the variance of local binary patterns (LBP) on the approximation subband (LL subband) generated by the stationary wavelet transform (SWT) of the test image. The work described in [20] presents another block-based CMFD method using cellular automata [21]. Techniques for describing image block features include discrete cosine transform (DCT) [22], Fourier transform (FT) [23], singular value decomposition (SVD) [24], and local binary pattern (LBP) [25]. However, most of these algorithms exhibit instability when applied to image geometric operations (such as rotation and scaling) or post-processing operations (such as JPEG compression, Gaussian noise addition, color restoration, scaling adjustment, brightness variation, and image blurring). A major drawback of these algorithms is their high computational load, primarily due to the need to compare a large number of image blocks. Kumar et al. [26] developed a method focused on minimizing computational requirements, particularly for large images with only a few features. The high execution time is a limitation of block-based algorithms; therefore, many researchers have adopted keypoint-based techniques to address these challenges. However, block-based CMFD methods tend to be unreliable in complex scenes, particularly when dealing with large homogeneous regions or extensive post-processing operations.

### 2.2. Keypoint-Based CMFD

Although most block-based techniques can accurately localize forged regions, they suffer from significant computational complexity due to the large number of blocks. Therefore, a keypoint-based approach is proposed in [27]. In this method, the image blockization step is omitted. Instead, local features derived from keypoints are used to analyze the image. Keypoints can be found in corners, edges, and regions of interest. Local features are extracted as a set of descriptors created in the vicinity of keypoints. To detect repeated regions, each descriptor is compared with others [27].

Keypoint-based CMFD techniques are generally classified into two categories: (1) methods based on Speeded-Up Robust Features (SURF) [28]; (2) methods based on Scale Invariant Feature Transform (SIFT) [29]. Since SURF technology is robust to geometric transformations as well as most preprocessing and postprocessing attacks, it can localize forged regions. Therefore, Bo et al. [30] proposed using SURF [28] for CMFD. Many scholars, in order to further improve detection and localization performance, have proposed additional SURF-based methods [31,32]. For example, Zhang et al. [32] proposed an algorithm that uses SURF to improve flat region detection by enhancing pixel contrast using Contrast Limited Adaptive Histogram Equalization (CLAHE), thereby preparing source images for the SURF detector to effectively reveal duplicated regions. Due to the scale invariance of SIFT descriptors, it can localize duplicated regions [33], addressing the scale issue; however, this algorithm fails to detect small-sized duplicated regions. Ardizzone et al. [34] proposed another SIFT-based approach, which performs poorly when detecting multiple duplicated regions. However, the main issue with these algorithms is the lack of keypoints, which leads to failure in detecting forgeries in small-sized images and, due to insufficient keypoints, hinders the localization and differentiation of duplicated regions. For these reasons, Alhaidery et al. [35] proposed an algorithm that combines block-based and keypoint-based techniques. Although keypoint-based methods reduce computational complexity, their detection performance strongly depends on the density and distribution of keypoints.

Although these traditional methods have made significant progress, they are not applicable under all conditions, as not all forged images employ a single method of forgery.

### 2.3. Deep Learning-Based CMFD

Traditional CMFD methods offer relatively acceptable performance; however, they are limited to handling only certain types of forgery, are unable to detect multiple forged regions simultaneously, suffer from high computational costs, and can only address a few types of attacks.

Deep learning-based methods, particularly CNNs, exhibit satisfactory performance in the field of machine vision, such as image classification, object detection, and image segmentation. Consequently, many CMFD works employ various deep learning algorithms. In [36], Li et al. developed a pairwise ranking and patch-matching technique to identify copy-move forgeries under scaling and rotation. Li et al. [37] described another CMFD and localization method, which employs a superboundary-to-pixel (s-BPD) segmentation technique to extract repeated image blocks. Deb et al. [38] introduced an integrated feature extraction and matching framework that combines AKAZE with SIFT algorithms. Recent CMFD techniques proposed by researchers, such as the FAAT forgery-aware adaptive transformer method by Liu et al. [9], demonstrate strong cross-domain generalization capabilities in addressing diverse forgery techniques. The KLMN method proposed in [10] combines knowledge distillation, multi-clue fusion, and forgery localization techniques, while enhancing model efficiency and deployability through lightweight design. Some CMFD methods that work by combining feature extraction, feature fusion, and decoding modules to generate binary masks are also popular techniques today. The ASCA-squeeze net method proposed in [13] forms a hybrid, efficient deep learning network by combining the Aquila Sine Cosine Algorithm with the Squeeze Net deep learning architecture. The TBFormer proposed by Liu et al. [14] is a forgery localization method based on a dual-branch transformer architecture utilizing both global and local features. In [15], the careful design of feature extraction and matching mechanisms, hierarchical discriminative structures, and efficient post-processing techniques in a lightweight high-precision network balances the trade-off between computational efficiency and detection accuracy in forgery detection tasks. To capture subtle changes in forged regions, Shi et al. [17] proposed a pixel-level global network, PL-GNet, which integrates fine-grained forgery detection and localization strategies. Despite the significant progress achieved by deep learning-based CMFD methods, their localization accuracy may still degrade under challenging conditions, such as scale variations, noise interference, or complex post-processing operations.

Building upon the aforementioned research, we propose a robust detection and localization approach to image copy-move forgeries using multi-feature fusion that is designed to overcome limitations of existing deep learning-based CMFD techniques. By comprehensively mining features across different image domains and leveraging inter-domain feature complementarity, our approach significantly enhances detection accuracy and model generalization capabilities. Furthermore, it demonstrates superior performance across diverse complex forgery scenarios while improving discriminative efficiency and feature utilization effectiveness.

## 3. Proposed Method

**Figure 2:**
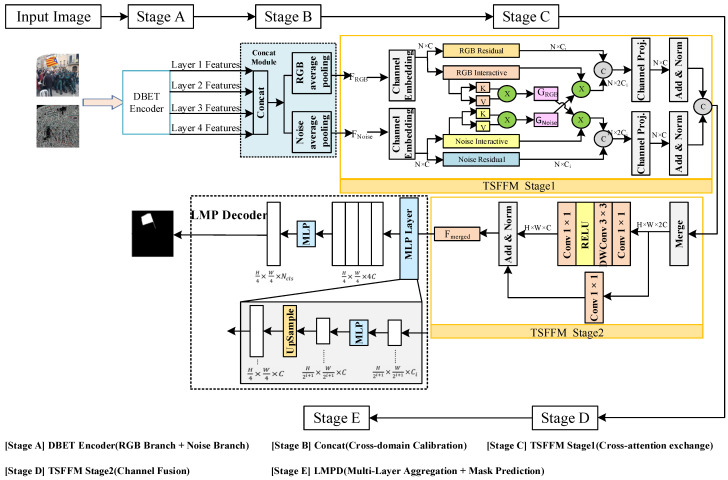
Overall architecture of the proposed MFFNet, illustrating the step-by-step processing pipeline from dual-domain feature extraction to multi-layer fusion and final localization. The DBET encoder extracts hierarchical features from RGB and noise domains, which are calibrated by the Concat module, exchanged through TSFFM Stage 1, fused by TSFFM Stage 2, and, finally, aggregated by the lightweight multi-layer perceptron decoder (LMPD) to generate the final prediction mask.

Figure 2 shows the architecture of the proposed MFFNet. The network consists of a DBET encoder, a Concat module, a two-stage feature fusion module, and a Lightweight Multilayer Perceptron Decoder (LMPD).

As shown in Figure 2, MFFNet is composed of four main components: the DBET encoder, the Concat module, the TSFFM, and the LMPD. The DBET encoder extracts discriminative features from both RGB and noise domains to capture complementary forensic cues. The Concat module connects the two feature branches and performs feature interaction and calibration through average pooling and reweighting operations. TSFFM consists of two successive phases, including an information exchange stage and a feature fusion stage, which facilitate global information interaction and multi-domain feature integration. Finally, LMPD utilizes hierarchical feature representations to reconstruct spatial details and generate pixel-level forgery localization results.

From an end-to-end perspective, the overall processing pipeline of MFFNet follows a clear and sequential information flow. The input image is first encoded by DBET to produce hierarchical representations from RGB and noise domains, enabling the extraction of both semantic inconsistencies and manipulation-related residual cues. These representations are subsequently calibrated by the Concat module to enhance cross-domain consistency. TSFFM then progressively refines the features by performing global information exchange via cross-attention and consolidating complementary cues through channel-level fusion. Based on the fused multi-layer representations, the lightweight MLP-based decoder aggregates contextual information and progressively upsamples the features to obtain the final forgery localization mask. This pipeline illustrates how multi-domain and multi-level features are jointly exploited to achieve accurate and robust copy-move forgery detection.

### 3.1. DBET Encoder

**Figure 3:**
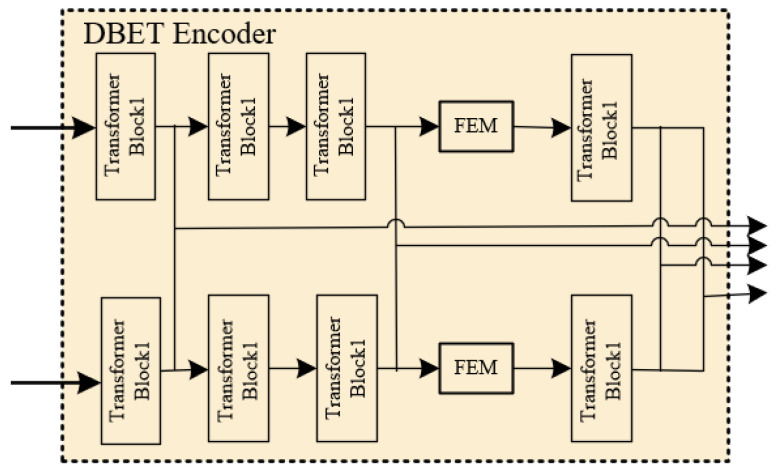
DBET encoder.

To leverage potential counterfeit cues from different domains, this paper employs two feature extraction branches, extracting discriminative features from both the RGB domain and the noise domain. These two branches have the same architecture but do not share weights, enabling them to focus on their respective domains. This paper employs CW-HPF [6] to convert the RGB domain into the noise domain. CW-HPF leverages inter-channel relationships of features and extracts noise features using a high-pass filter. Two types of attention modules are then applied on top of CW-HPF to model the internal dependencies in the spatial dimension and external dependencies between channels, utilizing a coarse-to-fine network to amplify the noise inconsistency between the original and tampered regions. The Transformer can overcome the limitation of CNNs, which have a limited receptive field, by modeling global contextual dependencies effectively. Rich contextual information is crucial for locating counterfeit regions; therefore, the feature extraction in DBET utilizes the Transformer. Figure 3 shows the detailed architecture of the encoder.

The input color RGB image $X\in R^{H\times W\times 3}$ is converted into a noise image $X^{\prime }\in R^{H\times W\times 9}$ using CW-HPF, where *W* and *H* represent the width and height of the input image, respectively. X and $X^{\prime }$ serve as the inputs to their corresponding feature extraction branches. X is divided into image patches of size 16 × 16, resulting in the sequence $A_{c}=\left\{a_{c}^{\left(1\right)},a_{c}^{\left(2\right)},\cdot \cdot \cdot ,a_{c}^{\left(S\right)}\right\}$, where $a_{c}^{\left(i\right)}\in R^{16\times 16\times 3}$ and S=H/16×W/16 represent the number of image patches. Each image patch $a_{c}^{\left(i\right)}$ is reconstructed into a one-dimensional vector, resulting in the image patch embedding sequence $P_{c}=\left\{p_{c}^{\left(1\right)},p_{c}^{\left(2\right)},\cdot \cdot \cdot ,p_{c}^{\left(S\right)}\right\}\in R^{D\times S}$, where D denotes the feature dimension. The positional embedding $pos_{c}^{\left(i\right)}$ corresponding to each position is added to the image patch embedding $p_{c}^{\left(i\right)}$, yielding the resulting input sequence $E_{c}=\left\{e_{c}^{\left(1\right)},e_{c}^{\left(2\right)},\cdot \cdot \cdot ,e_{c}^{\left(S\right)}\right\}\in R^{D\times S}$, where $e_{c}^{\left(i\right)}=p_{c}^{\left(i\right)}+pos_{c}^{\left(i\right)}$ denotes the final feature vector. $E_{c}$ is input into the feature extractor, constructed with four Transformer layers. The feature maps from the first, second, third, and fourth layers are output (i.e., $\left\{G_{c}^{\left(1\right)},G_{c}^{\left(2\right)},G_{c}^{\left(3\right)},G_{c}^{\left(4\right)}\right\}$):

(1) $$G_{c}=\left\{G_{c}^{\left(1\right)},G_{c}^{\left(2\right)},G_{c}^{\left(3\right)},G_{c}^{\left(4\right)}\right\}=f_{c}\left(E_{c}\right)$$

where $f_{c}$ denotes the feature extractor of the RGB branch.

The Transformer layer consists of multi-head self-attention (MSA) blocks and multi-layer perceptron (MLP) blocks. The architecture of the i layer can be represented as

(2) $$M_{c}^{\left(i\right)}=MSA_{c}^{\left(i\right)}\left(DS\left(G_{c}^{\left(i-1\right)}\right)\right)+G_{c}^{\left(i-1\right)}$$

(3) $$G_{c}^{\left(i\right)}=MLP_{c}^{\left(i\right)}\left(DS\left(M_{c}^{\left(i\right)}\right)\right)+M_{c}^{\left(i\right)}$$

where DS represents the layer normalization. The $MSA_{c}^{\left(i\right)}$ block consists of a self-attention (SA) operation:

(4) $$SA_{c}^{\left(i\right)}\left(G_{c}^{\left(i-1\right)}\right)=soft\max\left(Q_{c}^{\left(i\right)}{\left(K_{c}^{\left(i\right)}\right)}^{T}/\sqrt{L}\right)V_{c}^{\left(i\right)}$$

where the query, key, and value are computed as $Q_{c}^{\left(i\right)}=G_{c}^{\left(i-1\right)}W_{cQ}^{\left(i\right)}$, $K_{c}^{\left(i\right)}=G_{c}^{\left(i-1\right)}W_{cK}^{\left(i\right)}$, and $V_{c}^{\left(i\right)}=G_{c}^{\left(i-1\right)}W_{cV}^{\left(i\right)}$ and $W_{cQ}^{\left(i\right)}$, $W_{cK}^{\left(i\right)}$, and $W_{cV}^{\left(i\right)}$ are the learnable parameters of the three linear projection layers in the self-attention mechanism.

The same processing is applied to the noise image $X^{\prime }$, resulting in $E_{n}\in R^{D\times S}$. $E_{n}$ is fed into the feature extractor of the noise branch, yielding the noise feature map:

(5) $$F_{n}=\left\{F_{n}^{\left(1\right)},F_{n}^{\left(2\right)},F_{n}^{\left(3\right)},F_{n}^{\left(4\right)}\right\}=f_{n}\left(E_{n}\right)$$

where $f_{n}$ represents the feature extractor of the noise branch and $\left\{F_{n}^{\left(1\right)},F_{n}^{\left(2\right)},F_{n}^{\left(3\right)},F_{n}^{\left(4\right)}\right\}\in R^{D\times S}$ refers to the feature maps output by the 1st, 2nd, 3rd, and 4th layers of the Transformer.

The feature maps from the two branches and four levels, $\left\{G_{c}^{\left(1\right)},G_{c}^{\left(2\right)},G_{c}^{\left(3\right)},G_{c}^{\left(4\right)}\right\}$ and $\left\{F_{n}^{\left(1\right)},F_{n}^{\left(2\right)},F_{n}^{\left(3\right)},F_{n}^{\left(4\right)}\right\}$, are respectively denoted as $\left\{Z_{1},Z_{2},Z_{3},Z_{4}\right\}$.

In summary, the DBET encoder is designed to hierarchically extract complementary forensic representations from both RGB and noise domains. The RGB branch focuses on semantic and texture-level inconsistencies, while the noise branch emphasizes residual-based manipulation traces. Through multi-layer transformer encoding, DBET produces four levels of hierarchical feature maps for each domain, enabling the model to capture both local and global contextual cues. These multi-level features serve as the foundation for subsequent cross-domain interaction and fusion, providing rich and diverse representations for robust copy-move forgery localization.

### 3.2. Concat Module

The detailed structure of this module is shown in the Concat module in Figure 2, where the features from the two branches of the feature extractor are concatenated into $Z^{\prime }=\left[Z_{1},Z_{2},Z_{3},Z_{4}\right]$. The features are processed using average pooling in both the RGB and noise domains as follows:

(6) $$Z^{\prime }\left(r\right)=RAP\left(Z^{\prime }\right)$$

(7) $$Z^{\prime }\left(n\right)=NAP\left(Z^{\prime }\right)$$

where RAP refers to RGB average pooling and NAP refers to noise average pooling. In Equation (8), the spatial information of RGB and noise is encoded as T:

(8) $$T=sigmoid(BN\left(conv_{1\times 1}\left(\left[Z^{\prime }\left(r\right),Z^{\prime }\left(n\right)\right]\right)\right))$$

where $\left[Z^{\prime }\left(r\right),Z^{\prime }\left(n\right)\right]$ denotes the concatenation operation. The tensor T is split along the spatial dimension into two independent tensors, $T^{r}$ and $T^{n}$. These two tensors are flattened and used to generate the attention maps $M_{r}$ and $M_{n}$:

(9) $$M_{r}=sigmoid\left(conv_{1\times 1}\left(T^{r}\right)\right)$$

(10) $$M_{n}=sigmoid\left(conv_{1\times 1}\left(T^{n}\right)\right)$$

The attention maps are used to extract the interaction-augmented RGB features, $F_{RGB}$, and the noise features, $F_{Noise}$:

(11) $$F_{RGB}=Z^{\prime }\left(r\right)⊗M_{r}⊗M_{n}$$

(12) $$F_{Noise}=Z^{\prime }\left(n\right)⊗M_{n}⊗M_{r}$$

### 3.3. Two-Stage Feature Fusion Module

After obtaining the feature maps of each layer, TSFFM is constructed to enhance the interaction and combination of information. As shown in the TSFFM Stage 1 module in Figure 2, it represents the first phase of the feature fusion module, the information exchange phase. It maintains two branches and employs a cross-attention mechanism for global information exchange between them. As shown in the TSFFM Stage 2 module in Figure 2, it represents the second phase of the feature fusion module, the information fusion phase. The connected features are transformed back to their original dimensions through mixed-channel embeddings.

In the information exchange phase, bimodal features exchange their information through a symmetric, dual-path structure. For simplicity, the X-modal path is used for illustration. First, the input features of size $R^{H\times W\times C}$ are flattened into $R^{N\times C}$, where N=H×W.Then, linear embeddings are used to generate vectors of the same size as $R^{N\times C_{i}}$, referred to as the residual vector $X^{res}$ and the interaction vector $X^{\mathrm{int}er}$. An efficient cross-attention mechanism is further introduced, applied to the interaction vectors from the two different modality paths, which enables comprehensive information exchange across modalities. This provides complementary interaction from a sequence-to-sequence perspective, surpassing the correction-based interaction from the feature map perspective in CM-FRM. The cross-attention mechanism used to enhance cross-modal feature fusion is based on traditional self-attention [39]. The original self-attention operation encodes input vectors as $Query\left(Q\right)$, $Key\left(K\right)$, and $Value\left(V\right)$ and calculates the global attention map via matrix multiplication $QK^{T}$, which results in an $R^{N\times N}$ output and causes higher memory consumption. In contrast, a global context vector $G=K^{T}V$ of size $R^{C_{head}\times C_{head}}$ is used, and the attention result is calculated by QG. Based on this efficient self-attention mechanism, this redefinition was flexibly adapted to implement the multi-head cross-attention in this study. Specifically, the interaction vectors were embedded into K and V of each image, both of size $R^{N\times C_{head}}$. The output was obtained by multiplying the interaction vectors with the context vectors from the other modality path, i.e., the cross-attention process, which is described by the following Equations (13)–(16):

(13) $$G_{RGB}=K_{RGB}^{T}V_{RGB}$$

(14) $$G_{X}=K_{X}^{T}V_{X}$$

(15) $$U_{RGB}=X_{RGB}^{\mathrm{int}\mathrm{er}}SoftMax\left(G_{X}\right)$$

(16) $$U_{X}=X_{X}^{\mathrm{int}\mathrm{er}}SoftMax\left(G_{RGB}\right)$$

where G represents the global context vector and U represents the attended result. To enable attention from different representation subspaces, this study retained the multi-head mechanism, where the number of heads matched that of the Transformer backbone. Next, the resulting vector U and the residual vector $X^{res}$ are concatenated. A second linear embedding is applied, and the feature size is adjusted to $R^{H\times W\times C}$.

In the fusion stage, the second stage of the TSFFM, a simple channel embedding is used to merge the features from the two paths, which is achieved through a 1 × 1 convolutional layer. Additionally, during this channel fusion process, information from surrounding regions should be utilized to perform robust RGB−X segmentation. Therefore, a deep convolutional layer ($DWConv_{3\times 3}$) is added to implement a skip connection structure. The merged feature size is $R^{H\times W\times 2C}$, which is then fused into the final output of size $R^{H\times W\times C}$ for feature decoding.

### 3.4. Lightweight Multi-Layer Perceptron Decoder

The MFFNet architecture integrates a lightweight multi-layer perceptron (MLP) decoder, which avoids the handcrafted and computationally intensive components typically used in other methods. The key idea is to leverage Transformer-induced features, where the attention in the lower layers tends to remain local, while, in the higher layers, attention becomes highly non-local. The key to implementing this type of decoder is that the hierarchical transformer encoder has a larger effective receptive field (ERF) compared to traditional CNN encoders. The LMP decoder module in Figure 2 illustrates the detailed architecture of the decoder.

The LMPD consists of four main steps. First, the multi-domain features $F_{i}$ processed by the TSFFM module are passed through an MLP layer to unify the channel dimensions. In the second step, the features are upsampled by a factor of 1/4 and concatenated. In the third step, the concatenated features F are fused using an MLP layer. Finally, another MLP layer uses the fused features to predict the segmentation mask M, with a resolution of $\frac{H}{4}\times \frac{W}{4}\times N_{cls}$, where $N_{cls}$ represents the number of classes. The decoder is defined by the following Equations (17)–(20):

(17) $${\hat{F}}_{i}=Linear\left(C_{i},C\right)\left(F_{i}\right),\forall i$$

(18) $${\hat{F}}_{i}=Upsample\left(\frac{W}{4}\times \frac{W}{4}\right)\left({\hat{F}}_{i}\right),\forall i$$

(19) $$F=Linear\left(4C,C\right)\left(Concat\left({\hat{F}}_{i}\right)\right),\forall i$$

(20) $$M=Linear\left(C,N_{cls}\right)\left(F\right)$$

where M refers to the predicted mask, $Linear\left(C_{in},C_{out}\right)\left(\cdot \right)$ refers to a linear layer, and $C_{in}$ and $C_{out}$ represent the input and output vector dimensions, respectively.

Traditional CNNs are limited by the size of their receptive fields, which necessitate the use of contextual modules to expand them. However, this inevitably leads to heavier models. The decoder proposed in this work benefits from the non-local attention mechanism in Transformers, enabling the generation of larger receptive fields without increasing complexity, thus avoiding the handcrafted and computationally expensive components typically used in other methods, resulting in a lightweight model. However, the same decoder design does not perform well on CNN backbones, as the upper limit of the receptive field is constrained. More importantly, the decoder proposed here inherently leverages the features introduced by Transformers, which can simultaneously generate both highly local and non-local attention. By unifying these features, the LMPD decoder demonstrates complementarity and powerful representational capabilities with the addition of only a few parameters. Simply relying on non-local attention is insufficient to achieve optimal results, which is one of the key reasons for this design approach.

## 4. Experimental Results and Analysis

### 4.1. Experimental Datasets and Evaluation Metrics

#### 4.1.1. Experimental Datasets

This study analyzed and evaluated image forgery detection and localization methods using four publicly available datasets: CASIA v1 [40], COLUMB [41], NIST16 [42], and Fantastic Reality [43]. The CASIA v1 dataset contains splicing, copy-move, and removal forgeries. The forged regions have undergone careful manipulation and post-processing, including filtering and blurring, but the ground truth mask for the forged regions is not provided. The CASIA v1 dataset includes 5123 forged images in TIFF and JPEG formats. Most of the images have a resolution of 384 × 256. The COLUMB dataset contains 180 forged images in TIFF format, with typical resolutions ranging from 757 × 568 to 1152 × 768. This dataset only contains copy-move forgeries, with the forged regions consisting of large, meaningless areas. The corresponding ground truth masks are provided. The NIST16 dataset is a challenging dataset containing 564 forged images in JPEG format. It includes three types of local forgeries: splicing, copy-move, and deletion. The forged images in this dataset have undergone post-processing to hide any visible manipulation traces, and the corresponding ground truth masks are provided. The Fantastic Reality dataset is more extensive in terms of scene diversity and image quantity. It contains 16,000 forged images and 16,000 real images. This dataset includes splicing and copy-move forgeries and provides ground truth masks, instances, and class labels for each image.

The proposed method selected only the copy-move forged images from each dataset, as the goal was to detect copy-move forgeries and localize the forged regions. The CASIA v1 forged images were randomly split into 1350 images for training, 350 for validation, and 150 for testing. The ground truth masks for each image were manually crafted. The forged images from COLUMB were resized to 757 × 568 and randomly split into 150 images for training, 50 for validation, and 100 for testing. The forged images from NIST16 were randomly split into 200 images for training, 50 for validation, and 100 for testing. All images were resized to 512 × 384. The forged images from the Fantastic Reality dataset were randomly split into 10,800 images for training, 1200 for validation, and 1000 for testing. All images were resized to 512 × 512. Furthermore, various attack cases were created by applying JPEG compression, noise addition, and resizing operations to the four test datasets, as described below, to validate and assess the robustness of the detection method.

- JPEG Compression: The copy-move forged images were saved in JPEG format with different compression quality factors (*Q*).
- Noise Addition: Gaussian white noise with a mean of 0 and varying variances was added to the forged images.
- Resizing Operation: The forged images were scaled using a scaling factor.

Existing traditional detection methods are based on compression characteristics and can only detect JPEG-format images. Therefore, before conducting the comparative experiments, all TIFF-format experimental images were converted to JPEG format with a *Q* of 100%. The images used in the experimental section are listed in Table 1.

#### 4.1.2. Evaluation Metrics

The performance of the proposed method was evaluated using Precision, Recall, and F-measure. Precision is the ratio of correctly detected pixels to all detected pixels. Recall is the ratio of correctly detected pixels to ground truth pixels. F-measure is the weighted harmonic mean of Precision and Recall, combining both to provide a comprehensive evaluation of a model’s performance. In the subsequent experiments, Precision, Recall, and F-measure were averaged over all images in each case. The defining formulas are given by Equations (21)–(23):

(21) $$Precision=\frac{N_{TP}}{N_{TP}+N_{FP}}$$

(22) $$Recall=\frac{N_{TP}}{N_{TP}+N_{FN}}$$

(23) $$F=\frac{2\times Precision\times Recall}{Precision+Recall}$$

where $N_{TP}$ represents the number of forged pixels correctly classified, $N_{FP}$ represents the number of real pixels misclassified as forged, $N_{TN}$ represents the number of real pixels correctly classified, and $N_{FN}$ represents the number of forged pixels misclassified as real.

The proposed method only considered methods that could localize the forged areas at the pixel level. These included Local Binary Patterns (LBP) [25], Speeded-Up Robust Features (SURF) [28,44,45], KLMN [10], PL-GNet [17], MVSS [46], and PSCC-Net [47]. Some detection methods, such as those in [19], can only determine the type of forgery and were therefore excluded from this comparison. The first four methods are based on traditional classical techniques. The remaining four detection methods are CNN-based, and the hyperparameter values for each detection network were set to those that achieved optimal performance in the original papers.

### 4.2. Comparative Experiments and Analysis

#### 4.2.1. Experiments on Regular Forgery

In this section, the detection and localization performance of MFFNet and other competing detection methods in detecting regular copy-move forgeries is compared. The localization performance of MFFNet and other competing detection methods is evaluated using Precision, Recall, and F-measure, with the results presented in Table 2.

As can be seen from Table 2, compared to CNN-based detection methods, traditional methods exhibited lower accuracy and F-scores. SURF and LBP achieved exceptionally high recall rates, as they classified the entire image as a forged region. MFFNet outperformed other CNN-based detection methods on the CASIA v1, COLUMB, NIST16, and Fantastic Reality datasets. Additionally, MFFNet was compared with MVSS on the Fantastic Reality dataset, as MVSS required the class segmentation only provided by this dataset. It was particularly effective on the COLUMB dataset, possibly due to MFFNet’s use of a dual-branch fixed encoder for extracting structural information, which differentiates it from other methods. As a result, MFFNet effectively located large and meaningless forged regions. On the Fantastic Reality dataset, MFFNet’s improvement was relatively modest, likely due to the larger number of samples in the dataset, which allowed other methods to learn richer forgery cues. This further demonstrates MFFNet’s superiority in scenarios with limited samples. Clearly, among the compared methods, MFFNet achieved superior results, even when forged images contained multiple forged regions of varying scales.

#### 4.2.2. Experiments Under Various Attacks

To further validate the effectiveness and robustness of MFFNet, its performance was compared with that of other detection algorithms under attacks such as JPEG compression, noise addition, and resizing operations. Notably, none of the test sets used in the attack experiments were included in the training set.

**Figure 4:**
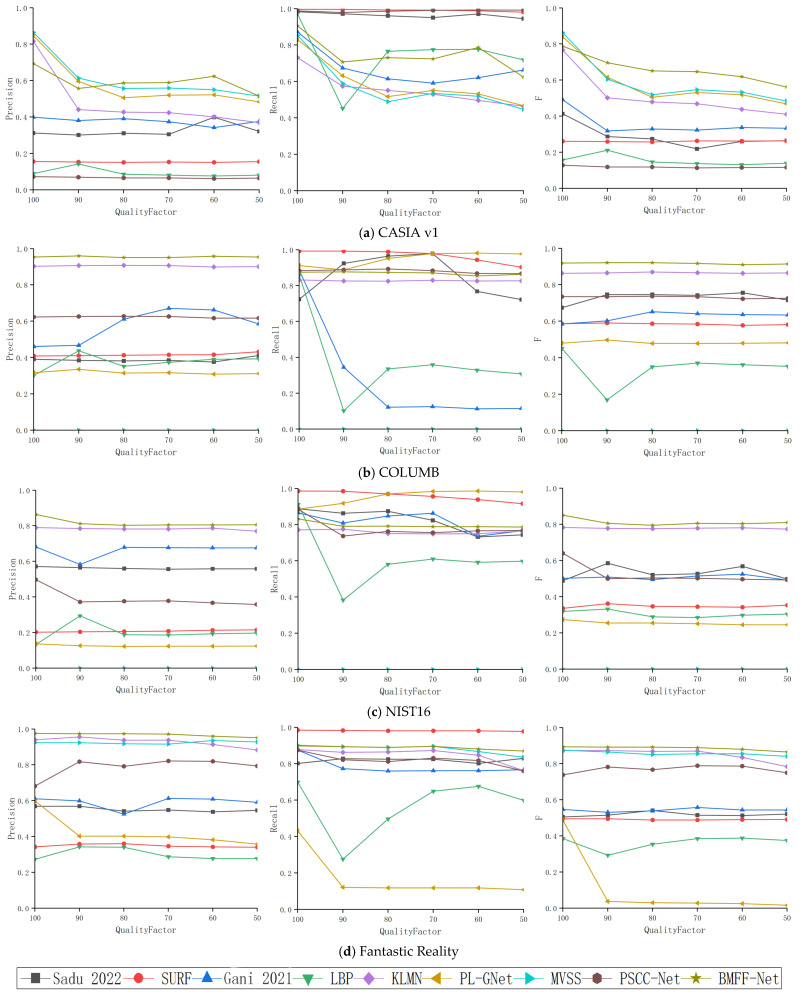
Experimental results under different JPEG compression levels [44,45]. (**a**) CASIA v1, (**b**) COLUMB, (**c**) NIST16, (**d**) Fantastic Reality.

Image compression is commonly encountered in daily life, as it is applied to most images on the internet and is a convenient method for concealing forgery traces. Therefore, comparative experiments were conducted under different JPEG compression levels in this study. The experimental results are shown in Figure 4.

As shown in Figure 4, the Precision, Recall, and F-measure of different detection methods were compared under varying JPEG compression levels for images from the CASIA v1, COLUMB, NIST16, and Fantastic Reality datasets. First, the experimental results indicate that JPEG compression affected the CASIA v1 images significantly, while its impact on the COLUMB, NIST16, and Fantastic Reality images was relatively minor. These results may be attributed to the different compositions of the datasets. The forged regions in COLUMB images are large and meaningless, whereas the NIST16 and Fantastic Reality datasets contain many samples forged from identical or highly similar images. The unique characteristics of the forged regions in these two datasets helped resist JPEG compression attacks. Secondly, as the quality factor decreased from 100 to 50, the Precision, Recall, and F-measure of most detection methods significantly declined, whereas MFFNet’s performance remained stable. At the same time, CNN-based detection methods achieved higher Precision and F-measure than traditional methods, with MFFNet outperforming other comparative methods. In this experiment, MFFNet demonstrated robust performance across all four datasets under various JPEG compression levels.

**Figure 5:**
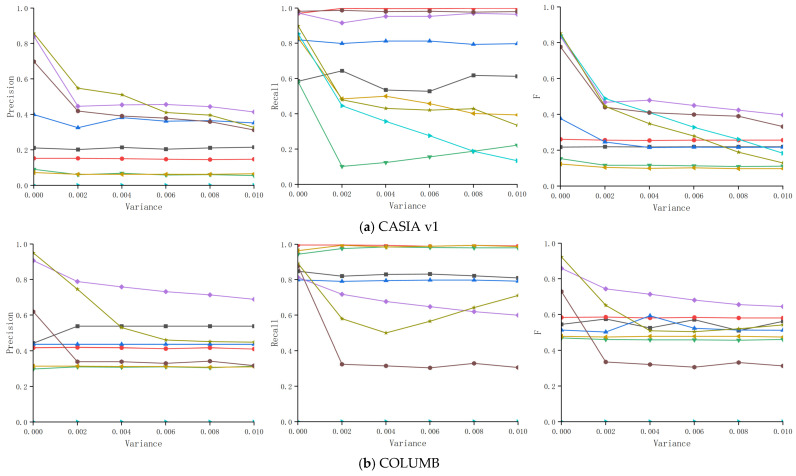

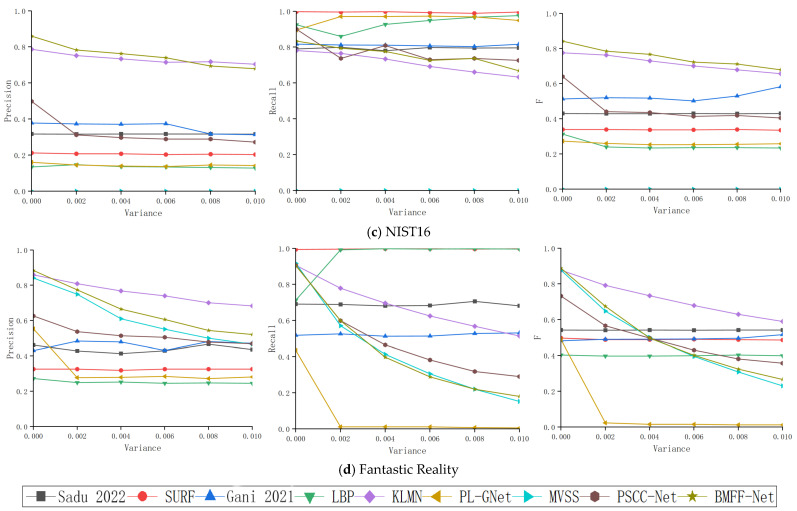
Experimental results under noise attack [44,45]. (**a**) CASIA v1, (**b**) COLUMB, (**c**) NIST16, (**d**) Fantastic Reality.

Adding noise to forged images is a common technique for concealing traces of image forgery. Therefore, an effective forgery detection method should exhibit sufficient robustness against added noise. In the comparative experiments, the robustness of MFFNet and other detection methods against noise was evaluated. The experimental results are shown in Figure 5.

As shown in Figure 5, the experimental results compare the Precision, Recall, and F-measure of images from the CASIA v1, COLUMB, NIST16, and Fantastic Reality datasets under added noise with varying variances. The detection performance of four traditional methods—LBP, SURF, and those from [44] and [45]—was nearly equivalent. Although their Precision and F-measure were lower than CNN-based methods, their Recall was higher, as these traditional methods tended to detect almost the entire image as the forged area. Among CNN-based detection methods, MFFNet achieved nearly the highest Precision and F-measure. However, it slightly outperformed KLMN on CASIA v1, COLUMB, and Fantastic Reality datasets, while MFFNet surpassed all methods on NIST16 in terms of both Precision and F-measure. This experiment demonstrates that the proposed MFFNet method exhibited excellent robustness against noise attacks across the four datasets.

**Figure 6:**
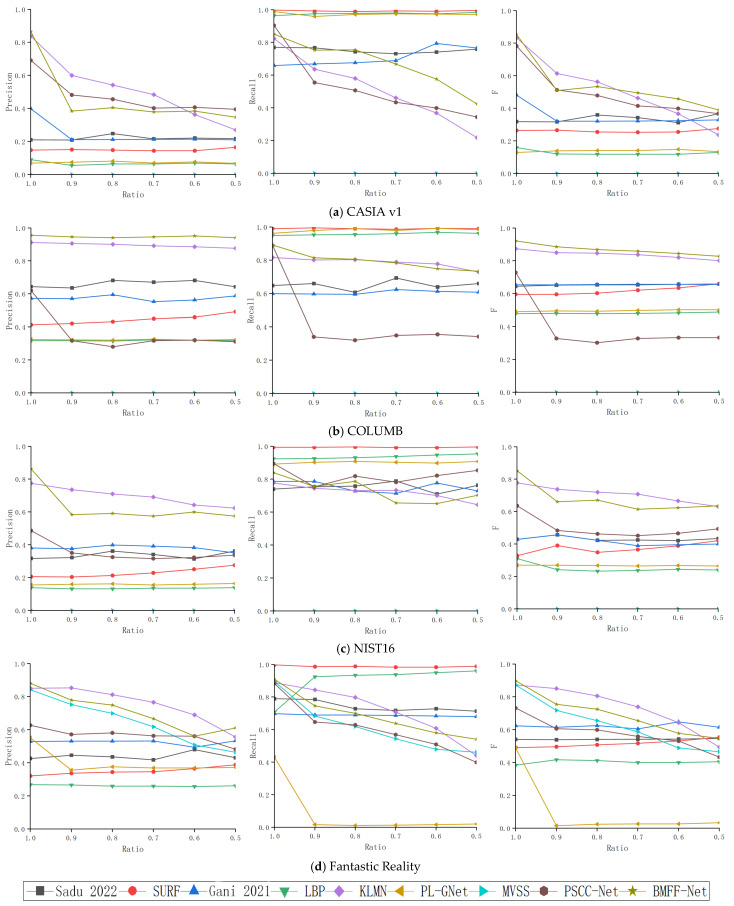
Experimental results under resizing attack [44,45]. (**a**) CASIA v1, (**b**) COLUMB, (**c**) NIST16, (**d**) Fantastic Reality.

Resizing provides another method for concealing forgery traces. Since resizing typically leads to some pixel loss, it increases the difficulty of detection. Figure 6 compares the performance of MFFNet and other detection methods under resizing attacks.

As shown in Figure 6, the Precision, Recall, and F1-score of various methods were compared under different scaling attacks across four benchmark datasets: CASIA v1, COLUMB, NIST16, and Fantastic Reality. Compared to most other methods, MFFNet achieved higher Precision and F-measure. However, KLMN performed slightly better on the CASIA v1, NIST16, and Fantastic Reality datasets. On the CASIA v1 dataset, MFFNet outperformed other detection methods overall, and it was only slightly lower than KLMN when the resizing ratio exceeded 0.8. At resizing ratios below 0.8, MFFNet consistently outperformed other detection methods. On resized COLUMB images, MFFNet achieved higher Precision and F-measure than existing methods. This experiment confirms the excellent robustness of the proposed model against resizing attacks across the four datasets.

### 4.3. Ablation Study

To evaluate the performance of the three main modules—DBET encoder, TSFFM, and LMPD—when combined, different variants were tested in a series of experiments conducted on the Fantastic Reality dataset. Table 3 shows the experimental results of the different variants. “RGB + LMPD” indicates that only the features from the final layer of the RGB branch were input into the decoder. “RGB + Noise + LMPD” indicates the use of the DBET encoder, but only the features from the final layers of both branches were simply concatenated and passed to the decoder. “RGB + Noise + TSFFM + LMPD” represents the proposed method, MFFNet.

As shown in Table 3, MFFNet provided more accurate detection and localization of forged regions, with each module contributing to the overall performance improvement of the model. By adding each module, both precision and recall were improved, leading to better accuracy in detecting forged regions with a low false positive rate; the model captured most forgery traces, such as edges of copied regions or noise consistency, while maintaining a low false negative rate. The increase in the F-score indicated that the model effectively balanced Precision and Recall, with no clear bias towards false positives or false negatives, thus ensuring overall good detection quality.

### 4.4. Visualization Analysis

**Figure 7:**
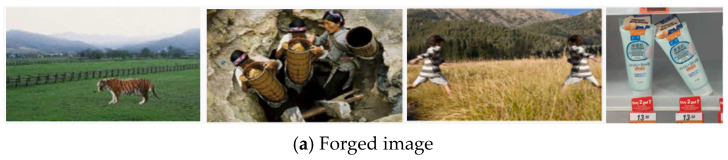

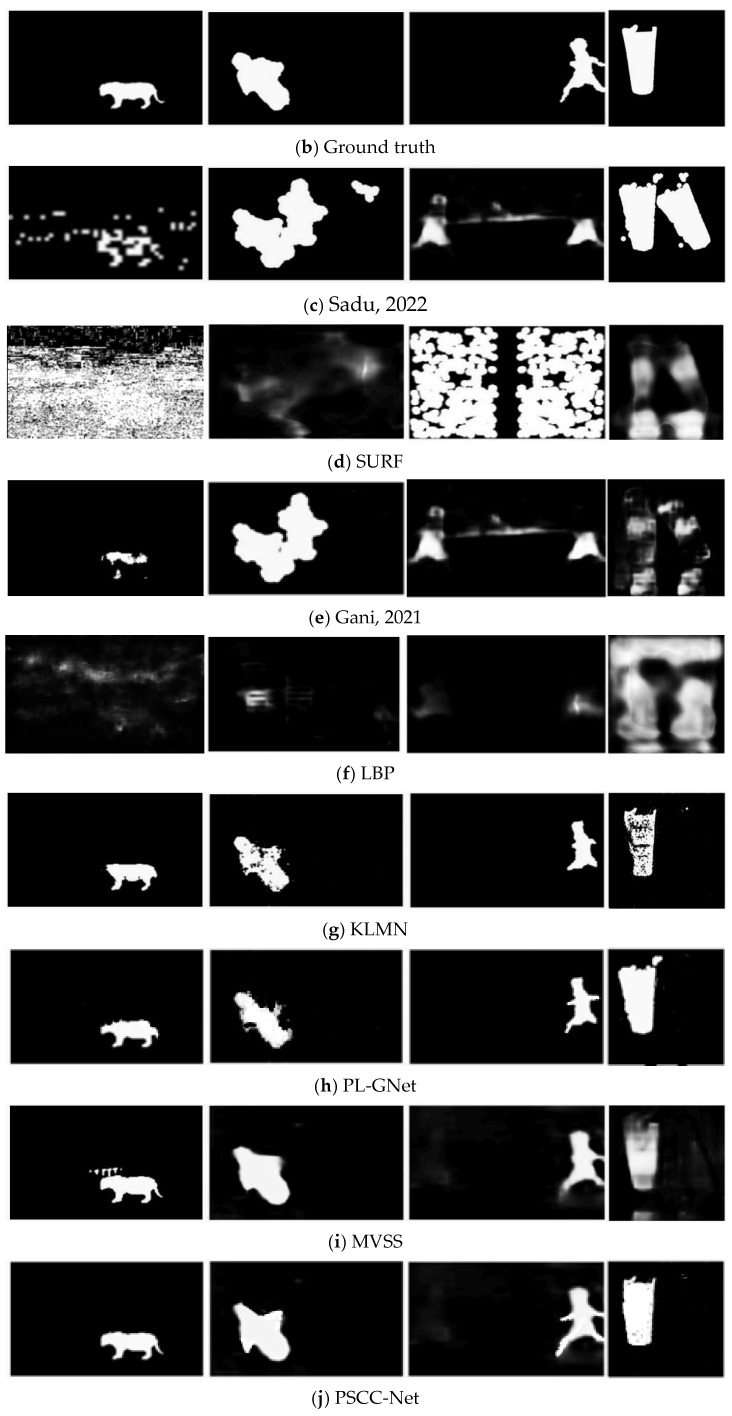

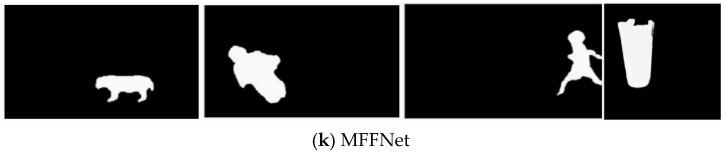
Visual comparison with various methods. (**a**) Forged image, (**b**) Ground truth, (**c**) Sadu, 2022 [44], (**d**) SURF, (**e**) Gani, 2021 [45], (**f**) LBP, (**g**) KLMN, (**h**) PL-GNet, (**i**) MVSS, (**j**) PSCC-Net, (**k**) MFFNet.

The comparative visualization in Figure 7 illustrates the forgery detection masks generated by the proposed MFFNet framework and other approaches across four standard evaluation datasets: CASIA v1 (first column), COLUMB (second column), NIST16 (third column), and Fantastic Reality (fourth column). Rows (a) and (b) correspond to the forged image and its corresponding real image, while rows (c)–(k) present the detection results of various methods.

As shown in Figure 7, the proposed MFFNet model achieved more precise forgery detection and localization across multiple scales compared to other methods.

### 4.5. Computational Complexity and Efficiency Discussion

Although the proposed MFFNet adopts a dual-branch Transformer-based architecture, its overall computational complexity is carefully controlled through a lightweight module design and efficient feature utilization. This subsection provides a qualitative analysis of the model’s computational cost, efficiency, and generalization ability, without modifying any experimental settings or evaluation metrics.

First, the dual-branch structure of the DBET encoder does not result in a proportional increase in computational burden. The RGB-domain and noise-domain branches share an identical architecture with a fixed number of Transformer layers, while focusing on complementary forensic cues from different domains. Such a design avoids excessive depth expansion and enables effective feature extraction without introducing redundant parameters. Moreover, the limited number of Transformer layers in each branch constrains both memory consumption and computational overhead. Second, the proposed two-stage feature fusion module (TSFFM) was designed with computational efficiency in mind. During the information exchange stage, the cross-attention mechanism employs a global context vector rather than a full attention matrix, thereby reducing memory usage compared with standard self-attention. In the subsequent fusion stage, channel embedding and depthwise convolution are utilized to integrate multi-domain features, which further limits parameter growth while preserving spatial contextual information. Third, the lightweight multi-layer perceptron decoder (LMPD) contributes to efficient mask generation. Unlike conventional CNN-based decoders that rely on heavy convolutional stacks or multi-scale context modules, LMPD consists only of MLP layers and simple upsampling operations. Benefiting from Transformer-encoded features with large effective receptive fields, the decoder aggregates multi-level information and produces accurate prediction masks with a relatively small number of parameters. This design reduces computational complexity in the decoding stage while maintaining localization accuracy. In addition, potential overfitting issues commonly associated with multi-branch Transformer architectures are alleviated through both architectural and experimental considerations. From an architectural perspective, the decoder remains lightweight and avoids over-parameterization. From an experimental perspective, the proposed model is trained and evaluated on multiple datasets with diverse image contents and attack scenarios, including JPEG compression, noise addition, and resizing operations. The consistent performance observed under these challenging conditions suggests that MFFNet exhibits robust generalization capability rather than dataset-specific overfitting.

Overall, MFFNet achieves a balanced trade-off between detection accuracy and computational efficiency. The proposed framework demonstrates that effective copy-move forgery detection and localization can be realized without resorting to excessively complex or computationally demanding architectures.

## 5. Conclusions

In this study, a multi-feature fusion framework, termed MFFNet, was proposed for robust copy-move forgery detection and localization. Extensive experimental results on multiple public benchmark datasets demonstrate that the proposed method consistently outperforms existing CMFD approaches in both detection accuracy and localization precision, particularly under challenging post-processing operations such as JPEG compression, noise addition, and image resizing. The superior performance of MFFNet is mainly attributed to two key design choices. First, the dual-branch DBET encoder effectively exploits complementary information from the RGB and noise domains, enabling more discriminative feature representations for copy-move forgery analysis. Second, the proposed two-stage feature fusion module facilitates efficient inter-domain interaction and multi-layer feature aggregation, which alleviates the limitations of insufficient feature fusion and weak decoding strategies observed in existing methods. In addition, the lightweight MLP-based decoder enables effective global context modeling without introducing excessive computational overhead, thereby supporting fine-grained forgery localization while maintaining model efficiency. The qualitative and quantitative evaluations further indicate that the proposed architecture exhibits strong robustness and generalization ability across diverse attack scenarios. Overall, the experimental evidence and analysis confirm that MFFNet provides a well-balanced solution for accurate, robust, and efficient copy-move forgery detection, making it a promising approach for practical image forensic applications.

## Figures and Tables

**Table 1:** Images used in the experimental section.

Name	/	Parameters	Range	Drop	CASIA v1	COLUMB	NIST16	Fantastic Reality
Training	/	/	/	/	1350	150	200	10,800
Validation	/	/	/	/	350	50	50	1200
Testing	General Forgery	/	/	/	150	100	100	1000
	JPEG Compression	*Q*	50–90	10	150 × 5	100 × 5	100 × 5	1000 × 5
	Noise Addition	Variance	0.002–0.01	0.002	150 × 5	100 × 5	100 × 5	1000 × 5
	Resizing Operation	Scale Factor	0.5–0.9	0.1	150 × 5	100 × 5	100 × 5	1000 × 5
All Images	/	/	/	/	4100	1800	1850	27,000

**Table 2:** Detection results for regular forgeries.

Method	CASIA v1			COLUMB			NIST16			Fantastic Reality		
	Precision	Recall	F	Precision	Recall	F	Precision	Recall	F	Precision	Recall	F
LBP	0.111	0.975	0.282	0.453	0.497	0.468	0.269	0.997	0.278	0.276	0.912	0.413
SURF	0.159	0.992	0.278	0.433	0.979	0.583	0.202	0.988	0.346	0.326	0.993	0.502
[44]	0.513	0.598	0.417	0.587	0.712	0.587	0.371	0.817	0.409	0.388	0.879	0.419
[45]	0.483	0.632	0.485	0.531	0.841	0.495	0.458	0.792	0.511	0.356	0.819	0.478
KLMN	0.858	0.836	0.856	0.908	0.833	0.877	0.791	0.790	0.782	0.859	0.891	0.875
PL-GNet	0.832	0.789	0.837	0.849	0.850	0.862	0.823	0.814	0.823	0.593	0.531	0.488
MVSS	/	/	/	/	/	/	/	/	/	0.835	0.916	0.875
PSCC-Net	0.795	0.928	0.798	0.635	0.889	0.731	0.511	0.898	0.641	0.666	0.848	0.756
MFFNet	0.869	0.865	0.861	0.958	0.911	0.929	0.878	0.839	0.867	0.879	0.922	0.893

**Table 3:** Experimental results for ordinary forgery.

Variant	Precision	Recall	F
RGB + LMPD	0.872	0.842	0.869
RGB + Noise + LMPD	0.912	0.895	0.899
RGB + Noise + TSFFM + LMPD	0.938	0.927	0.911

## Data Availability

The data presented in this study are available on request from the corresponding author due to privacy.
